# Innovative participatory evaluation methodologies to assess and sustain multilevel impacts of two community-based physical activity programs for women in Colombia

**DOI:** 10.1186/s12889-022-13180-2

**Published:** 2022-04-15

**Authors:** María Alejandra Rubio, Paula Guevara-Aladino, Marcela Urbano, Santiago Cabas, Carlos Mejia-Arbelaez, Patricia Rodriguez Espinosa, Lisa G. Rosas, Abby C. King, Scott Chazdon, Olga L. Sarmiento

**Affiliations:** 1 School of Medicine, Universidad de los Andes, Bogotá, Colombia; 2 Department of Epidemiology and Population Health, Stanford University School Medicine, Stanford, CA 94305 USA; 3 Division of Primary Care and Population Health, Stanford University School of Medicine, Stanford, CA 94305 USA; 4 Stanford Prevention Research Center, Department of Medicine, Stanford University School of Medicine, Stanford, CA USA; 5 University of Minnesota, Minneapolis, USA

**Keywords:** Physical activity, women’s health, Community-based programs, Breast cancer survivors, Behavioral intervention evaluation

## Abstract

**Background:**

Community-based physical activity (PA) programs are appealing to women in Latin America and show potential for improving women’s health. This study aimed to engage healthy middle-aged women, breast cancer survivors and local stakeholders participating in two publicly funded community-based PA programs in Bogotá, Colombia (*Recreovía* and *My Body*) to assess and visually map the perceived barriers, facilitators, and outcomes to promote programs’ improvement, scaling and sustainability.

**Methods:**

We used two participatory action research methods, the 1) *Our Voice* citizen science method to capture data and drive local change in built and social environmental facilitators and barriers that influence women’s engagement in community-based PA; and 2) Ripple Effects Mapping to visually map the intended and unintended outcomes of PA programs. We used thematic analysis to classify the results at the individual, social, and community levels.

**Results:**

The stakeholders engaged in the participatory evaluation included cross-sector actors from the programs (*N* = 6) and program users (total *N* = 34) from the two programs (*Recreovía N* = 16; *My Body N* = 18). Program users were women with a mean age of 55.7 years (SD = 8.03), 65% lived in low-income neighborhoods. They identified infrastructure as the main feature affecting PA, having both positive (e.g., appropriate facilities) and negative (e.g., poorly built areas for PA) effects. Regarding program improvements, stakeholders advocated for parks’ cleaning, safety, and appropriate use. The most highlighted outcomes were the expansion and strengthening of social bonds and the engagement in collective wellbeing, which leveraged some participants’ leadership skills for PA promotion strategies in their community. The facilitated dialogue among program users and stakeholders fostered the sustainability and expansion of the community-based PA programs, even during the COVID-19 pandemic.

**Conclusions:**

The implementation of both participatory methodologies provided a multidimensional understanding of the programs’ impacts and multisectoral dialogues that fostered efforts to sustain the community-based PA programs.

**Supplementary Information:**

The online version contains supplementary material available at 10.1186/s12889-022-13180-2.

## Introduction

The global trends of physical activity (PA) consistently show that women are less physically active than men (68.3% vs. 76.6%) [1]. This is critical for Latin America and, particularly Colombia, where only 43% of adult women meet the PA recommendations [2]. There is growing evidence that community-based PA programs are particularly attractive to women [3, 4]. Some studies have shown that more females than males and more older adults than any other age group use parks with community-based PA programs [5–7]. Given its acceptability among women of different ages and circumstances, some programs have been tailored to women with chronic conditions [8–11], including breast cancer survivors–a growing segment of the older women population [12]. In fact, evidence shows that dance-based programs have the potential to improve physical, emotional, and social aspects of breast cancer survivors’ health and wellbeing [8]. However, there is limited evidence concerning the actual extent and the level of benefits of community-based PA programs for women, both generally and for specific groups, and the mechanisms through which they may occur.

From a socioecological standpoint, PA is a behavior influenced by factors at multiple levels, including the individual (e.g., biological and psychological), social (e.g., sociocultural), and community (e.g., organizational, built environment, policy) levels [13]. Accordingly, women’s participation in PA is shaped by the physical and sociocultural environments impacting their daily lives [14–16]. Caregiving roles, expectations concerning daily household and work responsibilities, time management, and limited social support for PA are among the most well-documented influences reducing women’s PA [17]. In Colombia, gender inequities hindering women’s opportunities for education, employment, leisure, and personal care can negatively impact their PA levels [18]. To promote PA among women, programs need to address the multiple levels of the socioecological spectrum to successfully overcome the barriers that women face. Therefore, analyzing the context-specific conditions of women’s lives becomes urgent to better understand facilitators and barriers to PA, create equity and enhance women’s capability to manage their health and active lifestyles [19].

To expand our knowledge of how multi-level contexts influence women’s PA, it is important to use methodologies that adequately acknowledge individuals’ perspectives regarding such interacting influences and outcomes [20, 21]. Participatory action evaluations that capture multisectoral perspectives and impacts across levels of the socioecological model can uncover how community-based PA programs generate benefits beyond increasing women’s PA levels. Qualitative and participatory approaches such as the Our Voice citizen science model (OV) [22, 23] and Ripple Effects Mapping methodology (REM) [24] can reveal what factors across the socioecological spectrum facilitate or hinder PA for women, under what conditions, and for what reasons [25, 26]. The *OV* model [22] is a theory-based multi-sectoral citizen science intervention that allows local communities to collect real-world data on physical and social features that help or hinder their PA. Community members use their data to advocate for positive action steps in collaboration with local decision makers to develop supportive social, built, and political environments for PA [27]. *REM* [24] is a participatory evaluation strategy to facilitate the collective reflection among multi-sectoral stakeholders -including participants and others- in regard to the diverse impacts created by the intervention program, and the process by which the impacts came about. Working together, stakeholders visually map the broad outcomes that resulted from the community-based PA programs. Throughout this paper, we will use the term “stakeholder” to refer to public- and private-sector individuals with relevant community roles (policymakers, practitioners, consultants in public health and knowledge exchange experts) and interest in participating in the activities and decisions related to the community-based PA programs under investigation. Stakeholders included women and men. By engaging a variety of stakeholders, *OV* and *REM* can synergistically enhance knowledge and action steps for promoting PA among women.

Using two participatory methodologies (*OV* and *REM*), the overall goal of this study was to engage healthy middle-aged women, breast cancer survivors and other stakeholders to assess and visually map the multiple perceived barriers, facilitators, and outcomes of two community-based PA programs, and, ultimately, promote programs’ improvement, scaling and sustainability. The two Colombian PA programs being investigated were the *Recreovía* and *My Body*. The *Recreovía* is a public program, managed and funded by Bogotá’s Institute of Recreation and Sports, that offers free PA classes to the general public in local parks. *My Body* is a theory-driven intervention to promote PA through the *Recreovía* instructors among breast cancer survivors. Both PA programs (described below) have been evaluated using traditional approaches with objective measures such as standardized measurements for height, weight, PA levels [3, 28], quality of life [29] and sociodemographic characteristics of participants (age, socioeconomic level, education level). These evaluations have shown the potential of the programs to increase PA levels [3, 28]. However, the processes and outcomes surrounding both programs across levels of the socioecological framework have not been documented previously.

Specifically, we aimed to engage healthy middle-aged women, breast cancer survivors, and local stakeholders from both programs to take part in the *OV* model and the *REM* methodology to 1) advance program improvements by collectively identifying perceived built and social environmental facilitators and barriers that influence the maintenance of PA practice in urban settings; 2) visually map the intended and unintended outcomes at the individual, social, and community levels resulting from the engagement in the community-based PA programs; and 3) document community members’ and stakeholders’ sustainability efforts to continue promoting PA in their communities.

## Methods

### Setting and population

Bogotá, capital city of Colombia, has long shown a commitment to PA promotion through community-based strategies. Participants for this study were from two PA promotion studies in Bogotá: 1) the *Moving* study evaluated the *Recreovía* program occurring in one urban park in Bogotá across a sixteen-month period; 2) the *My Body* study (REF under review) was a theory-driven community-based pilot intervention promoting PA delivered by the Recreovía instructors among breast cancer survivors.

The *Recreovía* program is an initiative of Bogotá’s Institute of Recreation and Sports that for 25 years has offered free PA sessions, with components including Latin dance, aerobics, stretching, strength, and cardiovascular conditioning. The sessions are held in public spaces such as parks and plazas [3], and are open to community-wide members. Currently, the program offers classes on 95% of the city’s locality areas and users are likely to be women (80% overall attendance) with an average age of 49 years [30]. *Recreovía* has shown to be an effective strategy for promoting PA during leisure time in women [3]. Through a cross-sectoral collaboration, the *Recreovía-*trained instructors delivered a PA intervention for breast cancer survivors, *My Body*, co-created with academic researchers and stakeholders from the health sector. Participants were recruited according to *My Body* study eligibility criteria (i.e., women breast cancer survivors at least 6 months post completion of their treatment, more than 18 years of age, living in Bogotá, and willing to attend the program). The sessions were delivered in a community center managed by the *Recreovía* coordination and instructors. Because the vast majority of users attending each program were women, and, as noted earlier, women have been consistently shown to have lower PA rates than men, we focused specifically on the sample of women attending these programs.

### Study design

This study is framed using a socio-ecological framework relevant to PA [13] and grounded in social constructivism, defined as the acknowledgement of the human interactions through which meaning and knowledge are created [31]. To collect data, we used the following qualitative participatory methodologies able to capture participants’ perspectives:

The *OV* model empowers community members to identify local contextual factors impeding or facilitating regular PA and subsequently drive change in their local environments [23]. First, using the Stanford Healthy Neighborhood Discovery Tool mobile application, citizen scientists capture geocoded photographs and audio/text descriptions about negative or positive environmental aspects that influence their ability to lead healthy active lives [22]. Then, through facilitated community meetings held either in-person or remotely, citizen scientists review their data, discuss findings, prioritize aspects for change, and mobilize other residents and relevant stakeholders to promote improvements aimed at enhancing community health [32].

*REM* is a participatory evaluation method where participants and other stakeholders can visually map and, through a collective mind mapping and group interview process, uncover intended and unintended outcomes or “ripples” from the evaluated programs [24, 25]. During one researcher-facilitated group session, intervention participants and stakeholders work together to map the diverse “ripples” of the program by discussing program successes, unexpected outcomes, challenges, and solutions, and collectively organize insights by creating a map of all outcomes through a mind mapping software tool. Later, the research team summarizes the ripple effects maps and disseminates them to cross-sector stakeholders, as well as any appropriate public institutions with vested interest in the outcomes or the intervention.

All methods were carried out in accordance with relevant guidelines [33] and regulations, participants signed informed consent (to participate in the study and for publication of identifying images) and the study was reviewed and approved by the Universidad de los Andes ethics committee, Act Number 1258, 2020 (*Moving* study); 1251, 2020 (*My Body* study).

### Contextual data collection and analysis applying the Our Voice model

Between October and November 2019, participants from the *Recreovía* and *My Body* programs were invited via telephone to use the *OV* method’s Discovery Tool mobile application (app) to collect relevant contextual socioenvironmental data. Trained personnel provided participants with an Android mobile phone with the app installed. The data were uploaded via Wi-Fi to a secure Stanford University server.

1. *Community Walks: Recreovía* participants walked around the park environment after participating in the PA session. They identified the facilitators and barriers influencing regular attendance to the program using the Discovery Tool app. Participants of *My Body* (i.e., breast cancer survivors) scheduled their walks depending on their time availability and their preferred urban setting for engaging in PA after finishing the intervention.
2. *Facilitated community meetings to discuss data:* Two community meetings were held in November 2019, one for each program, with the aim of engaging citizen scientists in reviewing the data and prioritizing the findings. Two members of our research team conducted the meetings, which were audio taped and transcribed verbatim. For the *Recreovía* study, the meeting was held at the evaluated *Recreovía* park. The *My Body* community meeting took place at Universidad de los Andes’ facilities. At these meetings, citizen scientists reviewed their own data printed from the Discovery Tool App (verbatim transcriptions of each recorded narrative paired with the respective photographs and route maps). They shared with the group their impressions and visualized everyone’s insights by organizing the photographs on a display board, differentiating between facilitators or barriers, and identifying common themes. Subsequently, citizen scientists agreed on the three most relevant barriers to address and proposed solutions. Field notes from the community meetings were transcribed and analyzed to identify data-based categories. Finally, all the Discovery Tool transcripts were entered in an Excel spreadsheet (2016 version). Using a thematic analysis approach [34], two analysts independently coded the data based on the themes and sub-themes that emerged during the community meetings. In a second round, both analysts independently coded the data according to the socio-ecological framework to identify whether the facilitators and barriers corresponded to policy environment, built environment, social environment, or individual level of impact. In both rounds, two trained project staff members reviewed the coding and gathered in meetings to compare and validate the coded data, resolve inconsistencies, and obtain a final count of frequencies.
3. *Community meeting with stakeholders to promote community empowerment and change:* After the first community meeting, citizen scientists from each program gathered with stakeholders to discuss the prioritized barriers and feasible solutions. The meetings were conducted between February and March 2020 at the Universidad de los Andes.

### Evaluation using Ripple Effects Mapping methodology

Two *REM* sessions were conducted between February and March 2020 at the Universidad de los Andes, one for each program. Based on existing research and policy networks, stakeholders, citizen scientists, and family members or companions were invited. Each *REM* session took approximately 2 h and two members of the research team facilitated both sessions.

1. *Peer-to-peer interviews:* Participants broke up into groups of two and interviewed each other using an appreciative inquiry approach, a traditional interviewing technique within *REM* methodology [24, 35]. Questions were provided, focused on both achievements and challenges as a direct result of participating in each of the programs [24].
2. *Mapping the ripples:* Each participant reported the insights expressed during their peer-to-peer interviews to the larger group. One of the facilitators asked questions to probe deeper (e.g., “what activities led to that outcome?” “what happened after that?”), while the other facilitator recorded answers and started mapping using the XMind program [36]. The facilitators helped participants collectively organize their insights using the map and identify “labels” or themes for the common outcomes, as well as challenges and solutions. Lastly, participants shared final thoughts about their experiences.
3. *Systematic analysis of the maps:* After the *REM* sessions, two members of our research team streamlined the maps using the XMind program. Afterwards, the information from each map was exported to a table in Excel (2016 version). Two trained project staff members independently used the socio-ecological framework [13] to code each reported ripple as an individual, social, or community level outcome. Lastly, these two coders performed an intercoder reliability of the results [37], comparing their results to validate the coded data, resolve inconsistencies and obtain a final count of frequencies (total number of mentions) for each outcome.

### Sustainability efforts and community change

Our research team has established different channels to follow-up with participants in exploring what actions they have used for maintaining PA. By nurturing existing networks with stakeholders, we have been able to keep in contact with *Recreovía*’s coordination team and monitor the offered PA promotion programs. Consistent with participatory action methodologies, participants from both programs were invited to academic events to gain further information on methods used to maintain their PA. In addition, *My Body* breast cancer survivor were contacted through the WhatsApp group created during the intervention as a follow-up channel. In October 2020, phone calls to all *Recreovía* and *My Body* participants occurred by our study staff, asking about their PA behaviors after the study ended and during the COVID-19 pandemic.

## Results

### Stakeholders

The cross-sector local stakeholders that engaged in the participatory evaluation (*N* = 6) included one decision-maker, two physical activity instructors, one consultant in public health, and two clinical practitioners.

The *Recreovía* program participants (*N* = 16) were women on average 55.38 years old (SD = 8.78), 62.5% of them were married or living with a partner, 75% were from a low socioeconomic background, and 37.5% had a high school degree or less. Their mean body mass index (BMI) was 26.96 (SD = 3.46), and 94% of participants had a BMI categorized as overweight or more (i.e., ≥25).

For *My Body,* participants in the intervention arm were women (*N* = 18) with a mean age of 56 years (SD = 7.54), 44% of them were married or living with a partner, 56% were from low socioeconomic level, 56% had a school degree or less. Their mean BMI was 27.71 (SD = 4.71), and 61% of participants had a BMI categorized as overweight or obese.

### Findings from the *Our Voice* citizen science method

Overall, 12 participants engaged in the *Our Voice* process. A subsample of female users of the *Recreovía* program (*n* = 5) walked around the *Recreovía* park environment identifying facilitators and barriers to maintain PA practice. They collected a total of 41 photos and 40 comments through the Discovery Tool mobile app. Likewise, a subsample of breast cancer survivors from the intervention group in the *My Body* study (*n* = 7) evaluated urban settings available to help them maintain their PA participation after concluding the intervention. They used the Discovery Tool in their preferred urban setting to practice PA, which included three in local shopping centers, three in parks, and one in a community center. Overall, 45 photos and 45 comments were registered. Prior *Our Voice* studies have shown that similar or even smaller samples, 8–10 participants, are sufficient to achieve saturation and advocate for change [27].

Table 1 summarizes the local facilitators and barriers, according to the socio-ecological model, identified by the citizen scientists from both programs, discussed below. Complete *OV* findings are available in Additional file 1. (Our Voice: themes used to code Discovery Tool data per study and respective examples).

**Table 1:** Our Voice: Facilitators and barriers to physical activity from *Recreovía* and *My Body* studies

Program	Themes	Socioecological level	% per total of comments	Facilitator	Total # of comments per facilitator	Barrier	Total # of comments per barrier	Community meeting conclusions
**Recreovía**	**Recreovía program**	Policy	**8%**	Recreovía program	2		0	Cement floor is not a good choice for PA sessions, PA instructors will give posture indications to prevent injuries. To improve coexistence in the park citizen scientists suggested signposting promoting civic culture.
	**Infrastructure**	Built environment	**55%**	Outdoor fitness equipment	9	Lack of stage for Recreovía classes	6	
				Facilities improvement		Floor not suitable for AF		
				Disabilities inclusive facilities		Lack of illumination		
						Lack of a roof		
	**Park Logistics**			Park staff	3	Unused space	1	
				Space organization				
				Park hours				
	**Complementary services**				0	Toilets	1	
	**Diversity of activities**			Diversity of activities	2		0	
	**Access to nature**			Green areas	1			
	**Sense of community**	Social environment	**38%**	Support networks	4		0	
				Participation in different activities				
	**Sanitation maintenance**			Good waste management	1	Unsanitary portable toilets	2	
						Bad waste management		
	**Civic culture**				0	Sexual harassment in the park	4	
						Coexistence in shared space		
	**Safety**			Safety	2	Lack of safety	2	
**Total**			**100%**		**24**		**16**	
**My Body**	**Physical activity programs**	Policy	**4%**	Recreovía program	2		0	Periodic maintenance is required in order to prevent damage caused by regular use of facilities. Safety perceptions inhibits some citizen scientists from going to their nearby parks.
				PA programs offer				
	**Infrastructure**	Built environment	**62%**	Facilities	7	Poor facilities	6	
						Lack of facilities improvement		
	**Accessibility**			Easy access	4	Restricted transport options	2	
	**Nature**			Green areas	2	Air pollution	1	
	**Complementary services**			Bathrooms	3		0	
				Food supply				
	**Space logistics**			Diversity of spaces	1	Underutilization of spaces	3	
						Limited space		
	**Safety**	Social environment	**31%**	Safety	4	Lack of safety	1	
	**Sanitation maintenance**			Sanitation	1			
	**Medical condition**				0	Physical limitations	1	
						Lack of cancer-specific focus in programs		
	**Civic culture**			Co-existence at the park	1	Psychoactive substance use	3	
						Co-existence at the park		
	**Sense of community**			Support networks	2		0	
	**Self-determination**	Individual level	**2%**	Motivation for PA	1			
**Total**			**100%**		**28**		**17**	

#### Facilitators to community-based physical activity

Women from both studies identified facilitators and barriers mainly at the built environment level (59% of the comments), followed by social environment (35%) and policy environment (6%). Only women from *My Body* study evaluated local features at the individual level (2%) (see Table 1).

The most frequently mentioned facilitator in both programs was *infrastructure* (*Recreovía N* = 15/41, *My Body* = 13/45 photos), which included appropriate facilities to exercise (e.g., multipurpose community center and diverse sports courts). *Recreovía* participants highlighted the regular improvements and inclusive features of the park facilities for people with different levels of mobility. In addition, breast cancer survivors stated that comfortable facilities (clean, spacious closed area) improved the motivation to attend community-based PA programs.

For *Recreovía* participants, the second most relevant facilitator to PA was the *sense of community* fostered by the social network created by cohabiting the park, the program, and the neighborhood with other residents. In third place, they underscored *park logistics,* referring to the management of the park, considering space organization, staff, and park hours. Whereas in *My Body* study, breast cancer survivors identified *safety* (i.e., security staff and lightning) and *accessibility* (in terms of location and transport) as relevant aspects enabling women’s regular attendance to PA promoting urban settings. In words of citizen scientists:

*“I have lived in several neighborhoods, and this is such a humble but beautiful neighborhood because we have the best park in Bogota. I recreate myself; we all recreate ourselves and live happily because they give us a beautiful green field to do all the sport from 4:30 in the morning until we want to. That is the most beautiful recreation we have."* (*Recreovía* participant)

“*This is the entrance to the place* [community center] *where we do our exercises. It is a good area, very spacious, the floor is very safe, there’s good air flow and good lighting*.” (*My Body* participant)

#### Barriers to community-based physical activity

Regarding barriers, the one most mentioned in both studies was *inappropriate infrastructure,* concerning poorly built areas, lack of facilities’ improvements in parks, and the inadequate/hard surfaces for PA sessions (which generated knee pain). The second most mentioned aspect hindering participants’ PA and enjoyment of urban settings in both studies, was *limited civic culture*, a concept typically used to refer to the behavior of citizens in public spaces. Specifically, the participants of both studies mentioned the citizens’ poor management of dog waste. Additionally, this category contains one comment about sexual harassment in the *Recreovía* study, and comments about psychoactive substance use in parks in the *My Body* study. The third most mentioned barrier by *Recreovía* users was *poor sanitation maintenance* concerning unsanitary toilets and poor waste management. Whereas in third place, breast cancer survivors indicated *space logistics* issues, referring to underused spaces for potential PA practice [relevant for all evaluated urban settings].

One citizen scientist described: “*As you can see in the picture when you’re distracted you can fall down or hurt yourself because there’s a lack of maintenance of the park. A lot of people move around here and people on bikes as well which could also cause an accident.*” (*My Body* participant).

#### Advocacy process to foster program improvements

The community meeting for the *Recreovía* program was attended by the director of the program, two physical activity instructors, and five citizen scientists, while for *My Body*, the participants included one consultant in public health, two clinical practitioners, and six breast cancer survivors. In both meetings, citizen scientists shared their data with others who did not specifically collect these data. Upon discussing the most relevant issues to address, all attendants proposed solutions.

Safety and civic culture concerns were prioritized in both community meetings. In the *Recreovía* study, citizen scientists proposed expanding park cleaning and security staff to address the issues. Likewise, *My Body* citizen scientists proposed improving the parks’ safety with cameras, security staff, and public lighting. All participants noted that this would require more regular infrastructure maintenance to mitigate damage to the cameras and public lighting. In addition, women suggested implementing signage to mitigate the use of psychoactive substances/drugs (e.g., signage inviting to respect clean air) and to promote “civic culture” or shared values for the appropriate use of park settings. Faced with this issue, the Recreovía director agreed to discuss those concerns directly with the relevant decision-makers within the IDRD.

Additionally, to address the issue of the hard surfaces where the *Recreovía* sessions take place, based on the consensus built during the meeting, the *Recreovía* director agreed to ask the PA instructors to provide participants with lower-impact forms of PA, and posture guidance to help to reduce knee and joint pain.

On the other hand, citizen scientists of *My Body* study used the community meeting with stakeholders to highlight the *Recreovía* program as the main alternative to continue PA practice beyond the intervention. They celebrated the relevance of the cancer-specific focus integrated into *My Body’s* intervention, and *Recreovía’s* coordination decision to maintain the tailored sessions in the same site where the intervention took place. Some breast cancer survivors indicated that despite the distance/travel barrier, the main determining factors for continuing PA practice were PA-supportive infrastructure and safety.

### Findings from the Ripple Effects Mapping methodology

Tables 2 and 3 provide a summary of the ripple effects mapped from the *Recreovía* and *My Body* programs, respectively. The *Recreovía* REM session was attended by 18 program users, two family members, two physical activity instructors, and the program director. For *My Body* REM session attendants included 17 breast cancer survivors, two family members and friends, two clinical practitioners, and a consultant in public health. The review of the ripple effects map led to organizing the reported intended and unintended outcomes into the following: 1) *Recreovía* program: eight themes with 33 subthemes (Table 2, Additional file. 2), and 2) *My Body* program: nine themes and 42 subthemes (Table 3, Additional file. 2). Here we summarize the major outcomes noted in the sessions.

**Table 2:** Ripple Effects Mapping results from *Recreovía* study

Outcomes	Subthemes	Socio-ecological level				
		Ind.	Soc.	Com.	N	%
Expansion and strengthening of social bonds	Practicing physical activity with family	2	6	0	45	24
	Sharing new spaces with attendees		1	0		
	New social bonds	6	14	12		
	Possibility of doing physical activity with new people	1	3	0		
Awareness and engagement in collective wellbeing	Community appropriation and responsibility for the program	0	0	2	27	15
	Emerging leaders in the community	4	1	7		
	Developing spaces of community affection	2	1	3		
	Desire of sharing wellness with others	1	5	1		
Motivation for physical activity practice	Motivated by benefits of PA	3	2	0	27	15
	Motivated to have an active lifestyle	2	1	0		
	Motivated by enjoyment of PA	8	1	10		
Experiencing challenges	Discipline despite the circumstances	4	1	1	24	13
	Limited work with children and young adults	0	0	4		
	Shared use of parks	0	1	1		
	Taking advantage of the session for personal gain	1	2	2		
	Time management	4	0	1		
	Availability of session schedules	0	0	2		
Mental health	Well-being	5	4	2	23	12
	Quality of life	1	1	1		
	Stress management skills	3	0	0		
	Autonomy and self-care	6	0	0		
Program developments and improvements	Quality development of the Recreovía	0	1	2	16	9
	Trained staff	3	1	1		
	Reduction of inequities to access the physical activity opportunities	0	0	3		
	Increased amount of sessions	0	0	1		
	Appreciation of public institutions	0	0	4		
Physical health benefits	As a tool to deal with diseases and their treatments	2	1	2	13	7
	Positive effects on physical rehabilitation	6	1	0		
	Pain Management	1	0	0		
New physical activity habits	Expanding knowledge about healthy habits	2	0	0	10	5
	Interest in new forms of physical activity	4	1	0		
	Motor skill improvement	3	0	0		
**Total**		74	49	62	185	100

*Ind* individual level, *Soc* social level, *com* community level; N: total number of outcomes per themes

**Table 3:** Ripple Effects Mapping results from *My Body* study

Outcomes	Subthemes	Socio-ecological level				
		Ind.	Soc.	Com.	N	%
Expansion and strengthening of social bonds	New ties with institutions	2	1	2	37	19
	Enhancing family bonds	1	6	3		
	New social bonds	1	3	0		
	Reinforcing previous relationships	0	3	1		
	Sense of belonging among peers	2	5	0		
	Support network and peer-to-peer care	3	4	0		
Deepened physical activity engagement	Motivation for physical activity practice	1	1	0	26	14
	Regular physical activity practice	3	0	0		
	Interest in new forms of physical activity	3	6	4		
	Understanding physical activity as a therapeutic practice	3	1	0		
	Motivation because of the teachers	2	0	2		
Coping strategies for dealing with difficulties	Self-confidence to handle problems, obstacles and fears.	7	0	2	25	13
	Handling family setbacks	1	3	0		
	Shifts in family dynamics regarding caregiving duties	2	2	0		
	Changing risk perceptions	3	1	0		
	Changing self-efficacy perceptions	3	1	0		
Civic empowerment and leadership	Emerging leaders	2	3	4	25	13
	Desire to share well-being with others	1	2	0		
	Consolidation of a physical activity promotion network	1	2	1		
	Civic empowerment	0	1	8		
Experiences during the research	Appreciation of intersectoral work	1	2	2	22	11
	Gratefulness with the program facilitators	2	2	1		
	Consolidation of a friendly space	0	3	2		
	Impacts of research on the community	1	2	4		
Self-esteem	Sense of well-being	4	0	1	18	9
	Self-care as a priority	4	0	0		
	Further developing of resilience	4	1	0		
	Self-image perception	3	1	0		
Challenges for physical activity	Prioritizing other activities before personal care	1	1	0	16	8
	Accessing physical activity facilities	0	0	2		
	Risk perceptions and stigma	0	1	2		
	Socioeconomic barriers	2	0	0		
	Role as caregivers and the house chores	4	3	0		
Mental health benefits	Stress management skills	1	1	0	11	6
	Sense of tranquility	3	2	0		
	Overcoming the isolation	1	0	0		
	Social skills	1	2	0		
Physical health benefits	Weight control	2	0	0	12	6
	Pain reduction	2	0	0		
	Strengthening physical capacities	6	0	0		
	Sleep improvement	2	0	0		
**Total**		85	66	41	192	100

*Ind* individual level, *Soc* social level, *com* community level; N: total number of outcomes per themes

**Fig. 1:**
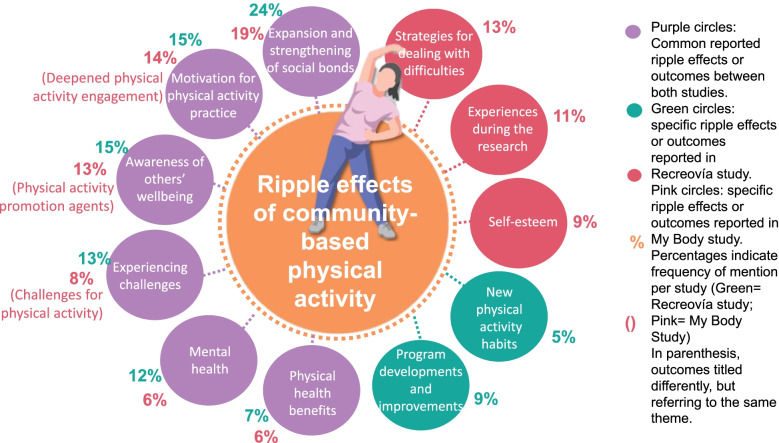
Ripple Effects Mapping: Results from Recreovía study and My Body study

In both studies, the most frequently mentioned theme was the *expansion and strengthening of social bonds* (Fig. 1). Participants reported that PA sessions acted as a place for a social gathering where they were able to enhance family bonds, reinforce previous relationships, and expand their social network, given the possibility offered by the community-based program of doing PA with new people. Particularly *My Body* program provided peer-to-peer support and a caring network, giving participants emotional support and a sense of belonging in the cancer survivor group.

One citizen scientist noted: “*I love this group with all my heart; it’s the biggest thing that The Lord has given me. I love being with them* [the other participants], *it’s a blessing. You get entangled amongst these wonderful people; we’ve become a family and you miss that family. I embrace that family, give them affection and a sense of safety. These are intangible things that can’t be said with words. I feel joy and satisfaction from being in this group. I’m happy, I feel joy and I feel motivated as a result of being a part of the group.”* (*Recreovía* participant).

Another participant noted: *“You can talk about chemo [therapy] freely, about breasts, about a lot of things that we have in common. This was very pleasant for me, to be able to be a part of a group”* (*My Body* participant).

For *Recreovía* users, the second most relevant outcome was *awareness and engagement in collective wellbeing*. Participants highlighted that the PA experience led to a desire to share wellness with others. According to participants’ narratives, experiencing a caring network within the program fostered community ownership and responsibility for the program, and developed and raised new leaders in the community. Similarly, breast cancer survivor from *My Body* intervention reported experiencing *civic empowerment and leadership* (third most frequent outcome) to advocate for PA and cancer care opportunities. For instance, when the intervention was over, participants submitted a right to petition (every citizen in Colombia has the right to present respectful petitions to the authorities on account of general or private interest and to secure its prompt resolution) to the IDRD asking to maintain the PA sessions with a cancer focus as part of the *Recreovía* program. The institution positively responded to this request.

One stakeholder noted: *“What caught my eye was that they [Recreovía users] would take the program to heart so that they became a fundamental part of its development. People explained that in the park, the neighbors would strive to get to their sessions; they’d bring the people together and involve them in the process. The program gave people meaning and has also greatly influenced not just their health but also the community around them.*” (*Recreovía* stakeholder).

A participant described: *“I always loved to dance and starting the classes was such a joy. Thanks to you, I can come back to the Clinic not just as a patient but as someone who can help other patients. I dance for them for two hours to Colombian music in the Chemo room, and I provide lectures on my experience with cancer to the patients and their caretakers. I was chosen as one of the leaders of the User League at the Clinic and I am taking classes to help not just the oncologists but also the people that come from out of town. Look at all that, where I am now, and to think I started out in the rumba sessions.”* (*My Body* participant).

Another outcome mentioned in both studies was *increased motivation for PA practice* (third most frequent theme in *Recreovía* study), also named in *My Body* study as *deepened PA engagement* (second most frequent theme). Some *Recreovía* users acknowledged that practicing PA with other participants enhanced their recognition of PA benefits (feeling relaxed, happy, vital), helped them to value their motives to have an active lifestyle (self-care, health management), and increased their enjoyment and satisfaction in doing PA. Similarly, breast cancer survivors stated having recognized PA as a pleasant habit for personal enjoyment. Indeed, a participant mentioned PA understanding and participation as a therapeutic practice. According to the women’s experiences, ongoing PA engagement was linked to increasing their overall motivation for regular PA practice and their interest in new forms of PA in addition to dancing. As citizen scientists mentioned: “*Exercise is life, health and it’s like a magic pill that you must take every day*” (*Recreovía* participant).

“*Honestly, I think that as of now I’ve seen changes. I think that for me this program was imperative and wonderful because it has seeded that habit within me. It’s true that now you feel the need to do it [physical activity] and doing it with others’ company is much better that going around the park by yourself*” (*My Body* participant).

Moreover, participants from *My Body* underscored as relevant outcome the use of *coping strategies for dealing with difficulties*. Breast cancer survivors expressed that by attending the program, they gained the self-confidence to overcome barriers that women often experience in practicing regular PA. For instance, family setbacks, household chores, and prioritization of caregiving activities. To handle some of these difficulties, they mentioned shifts in family dynamics regarding caregiving duties, and focusing on continuing to build their self-efficacy for PA. The participants mentioned that as the increased their self-knowledge of their bodies, they gained confidence and changed their risk perceptions concerning PA.

As noted by a participant: *“ I was able to get my siblings to become more involved in taking care of my parents and I was also able to get away from the monotony and the stress. It was a way for me to let go, delegate and spend some time on myself.”* (*My Body* participant).

Another participant noted that: *“The first two classes I never laughed. The instructor would ask “Where’s that smile?” and I’d just think “How do I laugh?” I didn’t even remember how to laugh. I’d get home, look in the mirror and say ‘How do you smile?’ That changed me a lot and opened up a whole new space as a human being within me.*” (*My Body* participant).

According to participants’ narratives in both studies, engaging in a community-based PA program enhanced wellness at all levels. They underscored that self-care was positively impacted through having a practice of personal enjoyment, leading to improved mental health, self-esteem, perceptions of well-being, better quality of life, and physical improvements. At the social level, they improved their social support and gained affectionate relationships. Particularly, breast cancer survivors mentioned experiencing social support from the intervention staff. Regarding the community level, all participants reported that engagement in the corresponding PA program created bonds with local public institutions.

As noted by a citizen scientist: *“I learned to love myself and make room for that space for me. Because normally, men are misogynists and for example I would go [to the Recreovía] every now and then when he wasn’t around. Other times I’d watch from my neighborhood and I couldn’t go because I had to make lunch. But no! I learned to make time for myself in that space. I learned to stop being submissive and defend something that’s mine. I owe that to the Recreovía.”* (*Recreovía* participant).

### Findings from the 9-month follow-up

After concluding these phases, our research team followed up concerning participants’ and stakeholders’ actions to continue promoting PA in their communities. With respect to stakeholder ongoing actions, *Recreovía* has implemented at least two new strategies to continue promoting PA during the COVID-19 pandemic. They have offered “La Ruta del Movimiento” which provides free PA sessions led by trained instructors in outdoor locations such as the communal areas of apartment buildings, enabling residents to see them from their windows, balconies, and terraces and participate at a safe social distance [38]. In addition, they currently offer PA sessions through Facebook live streaming. Overall, a total of 30 participants from both studies (*Recreovía n* = 14; *My Body n* = 16) completed the follow-up assessment. Of them, 63% knew about the *Recreovía*’s adaptations during the pandemic, and 47% of participants had engaged in the Facebook Live sessions.

All *Recreovía* participants reported continuing their PA practice after concluding the study and prior to the COVID-19 pandemic, and 71% of them continued their PA practice during the COVID-19 pandemic. The reasons why the others stopped during the COVID-19 pandemic included health, quarantine, fear of infection, and work. From the *My Body* study, 75% of participants reported continuing PA after the study was over, as well as during at least the first 9 months of the COVID-19 pandemic. For the four participants that stopped doing PA, the reasons were health, cancer-related complications, and limited space in their homes.

Through the *My Body* WhatsApp group, some participants took initiative to keep promoting PA by sharing dancing sessions and events. Additionally, participants from both studies have attended virtual academic events organized by our research team, aimed at leveraging continued dialogues between citizens and stakeholders.

## Discussion

Through a participatory action approach, this study successfully documented the perceived facilitators, barriers, and outcomes from two community-based PA programs mainly benefiting healthy middle-aged women and breast cancer survivors in a middle-income country. The implementation of both methodologies using the socioecological framework provided a multidimensional understanding of the programs [39] and multisectoral dialogues that fostered efforts to sustain the community-based PA programs. Findings from the *OV* method indicated that for both groups, *infrastructure* was the most mentioned aspect for the maintenance of PA practice in urban settings, performing both as a facilitator (e.g., appropriate facilities to exercise) and a barrier (e.g., poorly built areas for PA). Also, *civic culture* and *safety* were underscored as key social environmental factors for the enjoyment of PA. Among the potential program improvements, stakeholders prioritized measures to improve parks’ cleaning, safety, and appropriate use. The *REM* showed that the *expansion and strengthening of social bonds* was the most mentioned ripple effect among both programs. The engagement in collective wellbeing even leveraged some participants’ leadership skills to engage further in PA promotion strategies in their community. Ultimately, the facilitated encounters among program users and stakeholders enabled the celebration of such broad impacts of the PA programs and engagement for further PA maintenance, even during the COVID-19 pandemic.

Our study corroborates previous *OV* findings highlighting physical facilities as the main enablers, and limited infrastructure, as the most common obstacle to be physically active [22, 26, 40, 41] However, the relevance of these aspects varied between the two studies, reiterating the relevance of population and location-specific evaluations in order to tailor programs to local contexts and needs. The *Recreovía* study findings showed that the park was perceived as an appropriate environment for PA, with potential improvements regarding sanitation, maintenance, safety, signage, and park logistics. In contrast, the breast cancer survivors participating in the *My Body* study expressed that, given their perceived need for cancer-specific PA programs, finding urban settings that could provide safe environments for them to regularly engage in PA was challenging. Nonetheless, they underscored as facilitators the PA sessions offered in shopping centers, parks, and community centers, which are facilities that can provide proper infrastructure, safety, and accessibility. Our results are consistent with *Our Voice’s* overarching purpose to set in motion a self-sustaining process of citizen science where residents become aware of their ability to leverage urban settings and create a “culture” of active living, health, and well-being [22, 27, 41]. Participants from both studies have shown engagement to encourage active lifestyles, foster ongoing program sustainability, and disseminate the work to other relevant problem areas and communities [22, 26, 41, 42].

The implementation of the *REM* allowed stakeholders to further uncover and celebrate outcomes from the PA programs which benefitted themselves, their communities, and their environment. The findings add to the literature on the relationship between social capital and positive health outcomes indicating that community-based PA programs, as social interventions, use the social structures as drivers to support PA behaviors, enabling women to access and mobilize social capital for improving their health [42, 43]. In both of our studies, the interactive group discussions and reflection allowed women to express the ways in which participating in regular community-based PA strengthened social bonds, fostered the engagement in collective wellbeing, increased motivation to be physically active, and deepened PA engagement, among other outcomes. In addition, throughout the COVID-19 pandemic, 73% of participants across the two studies continued their PA practice through *Recreovía* strategies or self-managed groups, which is promising.

Some of the contextual barriers reported by participants from *Recreovía* and *My Body* have been reported in other studies involving PA and women, such as time management, caregiving roles, and safety; generally, caregiving roles have been reported often concerning child care [17, 44, 45]. however, in our study women mentioned it regarding elderly care. We learned that for both study groups, the expansion and strengthening of social bonds is a valuable outcome as it enabled further dynamics relevant to overcoming gender role constraints related to PA (e.g., family caregiving). Such dynamics included acquiring coping strategies to deal with difficulties and raising awareness of ways to expand PA motivation through their social networks. This is in line with previous evidence indicating that social support from family and friends is positively related to initiating and maintaining PA, particularly among Latina women [17, 46] Ultimately, recognizing how gender norms influence PA practice is essential to respond to women’s health needs and to improve programs [47, 48]. This adds to the social capital and health literature call to understand how social connections transfer into assistance for health-related issues [49].

Finally, our findings reveal the interconnectedness nature of the multi-level outcomes stemming from community-based PA programs and how these can rebound in program sustainability: as women engage in PA of personal enjoyment -feeling safe and supported-, their raised awareness towards self-care and increased motivation to be active expand to experiencing civic empowerment and advocating for collective wellbeing. Altogether this leads to PA maintenance at individual and program level, possibly indicating the relevance of including a “leadership skills strengthening” component in the PA interventions, as we never know who might be compelled to become a PA promotion agent. As reported by the participating stakeholders, the acknowledging of these rippled outcomes within multisectoral dialogues fosters efforts to implement and sustain community-based PA programs. Sustainability and scaling up of the programs is a never ending top-down, bottom-up synergy [26, 30].

### Limitations

The study findings must be seen in the light of some methodological limitations. The first is a possible selection bias given that participants were recruited during their participation in the targeted community-based programs. Therefore, this may show a generally high acceptance of the programs. Future studies should consider efforts to include those who refuse to engage or who have dropped out these programs to provide further information on program barriers to participation in particular. Second, a relatively small number of participants supplied contextual data through the use of the Discovery Tool app and the community meetings had limited participation from relevant decision-makers, which may limit program advocacy occurring for positive changes. Nonetheless, some tangible outcomes were produced through this participatory action process, including the ongoing dialogues between the IDRD and researchers to advise on the strategies within the COVID-19 pandemic. It would be worthwhile to continue to explore how community meetings could engage higher levels of participation from multi-sectoral stakeholders.

## Conclusion

By promoting women’s voices as the central target for engaging women in systematic community participatory action activities, this first-generation study revealed how community-based PA programs can contribute to addressing some of the barriers that women with different social and health conditions face to engage in PA [50, 51] and improve their health [44]. Successes of our investigation included the use of participatory evaluation methods with women and enabling stakeholders to uncover and celebrate the broad impacts of PA programs benefitting different groups of women. The study revealed that the impacts of the community-based PA programs can go beyond the participants’ regular practice of PA. At the individual level, they can develop a self-care practice of personal enjoyment, leading to improved self-efficacy, mental health, and perceptions of well-being. At the social level, they can improve their social capital and increase social connection. At the community level, participating women strengthened bonds with local public institutions, and some promoted the benefits of PA in their communities. Furthermore, our multisectoral approach established an ongoing dialogue with stakeholders, which can facilitate tangible changes in the local social and physical environmental contexts impacting women’s PA. The participatory action approach using the OV model and accompanying REM outcomes assessment method has the potential to leverage both community members’ and stakeholders’ motivations to maintain and disseminate the programs, as well as to continue to monitor the broader, multi-level impacts that can result from such participatory action methods.

## Supplementary Information

**Additional file 1: Supplementary material 1.** Our Voice results: themes used to code Discovery Tool data per study and respective examples.

**Additional file 2: Supplementary material 2.** Ripple Effects Maps created by participants to report outcomes of the Recreovìa and My Body physical activity programs, respectively.

## Data Availability

The datasets used and/or analysed during the current study are available from the corresponding author on reasonable request.

## Abbreviations

PA: Physical activity
OV: Our Voice citizen science model
REM: Ripple Effects Mapping
IDRD: Bogotá’s Institute of Recreation and Sports (Instituto Distrital de Recreación y Deporte)
IRB: Institutional Review Board
BMI: Body mass index
