# Supplementary material for: Innovative participatory evaluation methodologies to assess and sustain multilevel impacts of two community-based physical activity programs for women in Colombia
Source: BMC Public Health. 2022 Apr 15;22:771. doi: 10.1186/s12889-022-13180-2 (PMC9012256; doi:10.1186/s12889-022-13180-2)
Supplement: Supplementary file 1 — Additional file 1: Supplementary material 1. Our Voice results: themes used to code Discovery Tool data per study and respective examples. [file 12889_2022_13180_MOESM1_ESM.docx]

Supplementary material 1. Our Voice results: themes used to code Discovery Tool data per study and respective examples.

| Theme | Discovery Tool Narratives | Discovery Tool Photographs |
| --- | --- | --- |
| *Moving Study* |  |  |
| Infrastructure | "Well, in this picture, we can see one of the biggest projects that this park has right now, so it looks great [Multi-purpose building under construction]. There are going to be swimming pools, places where you can play sports, gyms, basketball courts. Well, according to what I have more or less realized, it seems great. I wish they would adopt more places like this. People need sport, a lot of physical activity. It's something that allows us to live better. Thank you." (Moving participant) | 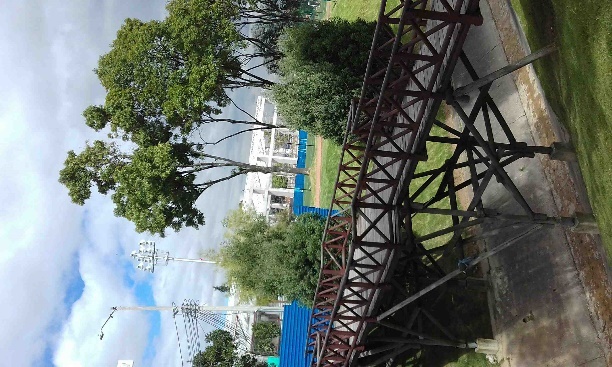 |
| Diversity of activities | "[This park] has several courts. There are basketball courts, soccer fields, there are some synthetic courts, which are rented at night when you see people playing. They are very well-lit." (Moving participant) | 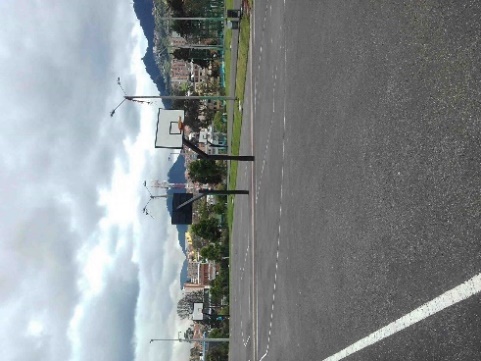 |
| Sense of community | "I am at the community center, very satisfied because the group is large to do the exercises. I feel very comfortable, the group is very helpful, we are very united, and I like it. Well, I am really happy and satisfied with this group and even more so with the IDRD teachers, who are very nice to us and keep us recreated and active. Thank you very much. " (Moving participant) | 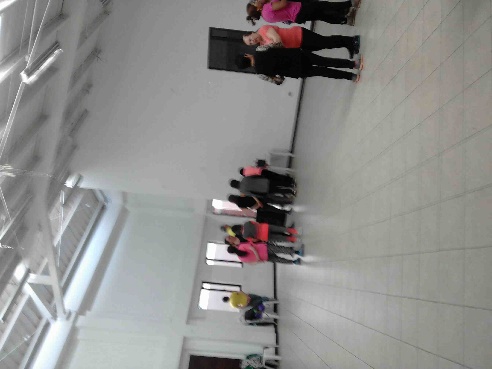 |
| Recreovía | "I take the picture because I think this is great. I think it's a gift, a gift the mayor's office gave us. I think all my neighbors and I take advantage of it. We have great teachers; for me, they are all great. My neighbors are great, wonderful. Above all, they are giving us health, and we thank God for having this recreation." (Moving participant) | 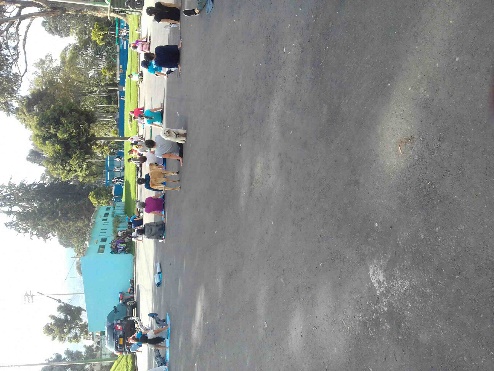 |
| Access to nature | "I have lived in several neighborhoods, and this is such a humble but beautiful neighborhood because we have the best park in Bogota. I recreate myself, we all recreate ourselves and live happily because they give us a beautiful green field to do all the sport from 4:30 in the morning until we want to. That is the most beautiful recreation we have." (Moving participant) | 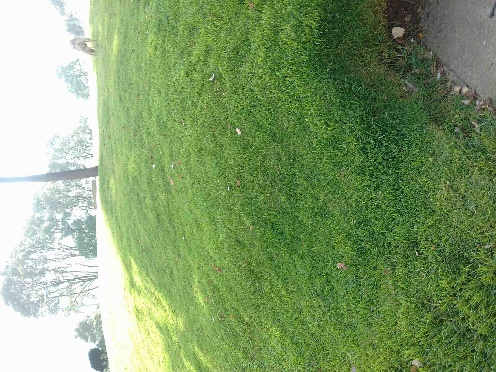 |
| Sanitation Maintenance | "Well, that other picture I took is because I like the cleanliness inside the park. The garbage disposals are very important because everyone brings something to eat or something, and there are garbage cans. I don't think it's difficult for anyone to get close and throw their waste into the garbage cans. I think that's very cool. " (Moving participant) | 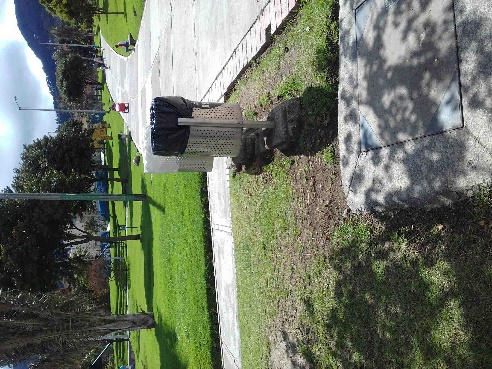 |
| Safety | "For such a big park, there is little security, not enough staff, no guards. I don't know if the cameras are working, but security for all users is very important." (Moving participant) | 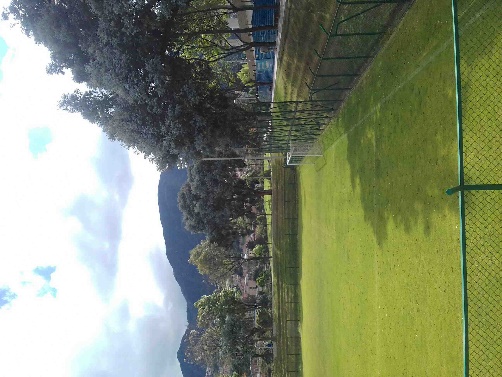 |
| Park logistics | "Well, here we can see 4 mini-football fields. Additionally, in the whole park, there are 2 other soccer fields, which are bigger. So, as we can see, there are many soccer fields, I think we could see if we could do other activities on those fields for other kinds of sports that people practice." (Moving participant) | 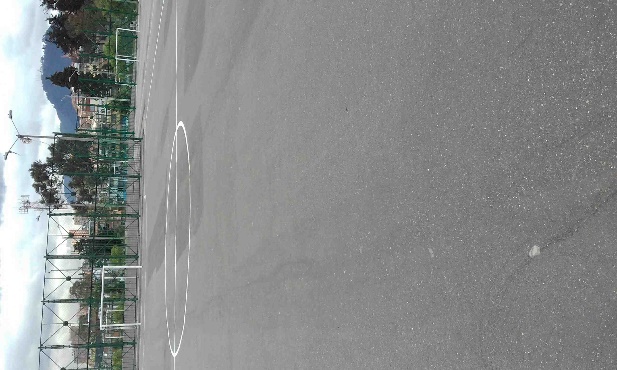 |
| Complementary services | "We are grateful for what we have so far. Let's not demand more because they're not going to put the roof on. I would like, please, I don't know if you can get them to lend us the room on rainy days. In that, we do ask for a collaboration, please. Don't leave us wet, washed, and the teachers stiff. Please lend us the room and if we have to clean it, well, as a group we'll clean it. We accept this collaboration temporally everything is provisional because we are going to enjoy this beautiful park. Thank you, everything for the mayor's office. Thank you." (Moving participant). |  |
| Civic culture | "... I hope they pay more attention to it, especially to the grooming of the dogs, because dogs are very dirty, and in that sense, I repeat, children cannot even come to sit in the park. Please, listen to us about this, let's give hours of recreation also to the little animals, the little animals have to recreate, too. But we have to recreate ourselves too, please. There are very heavy people when they are running, and the dogs get in their way, and they say, "Oh, stupid old man!” And the problem is that you are rude. So, pay attention to this, and that the guards are more aware of our park and rude people. Thank you." (Moving participant) | 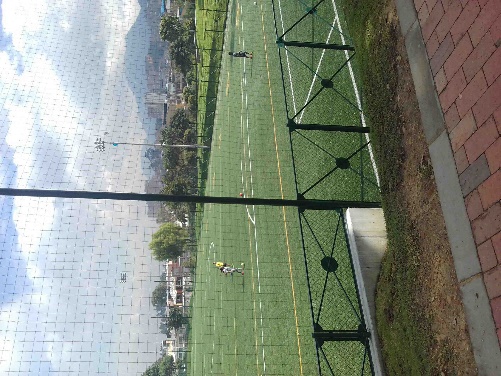 |
| *My Body study* |  |  |
| Infrastructure | "This is the room where we exercise. One of the barriers I see is that there are no windows, no good ventilation. Then Monday and Tuesday that we do the exercises, it fills up. Today, Saturday, there are few people. So we need more ventilation." (My Body participant) | 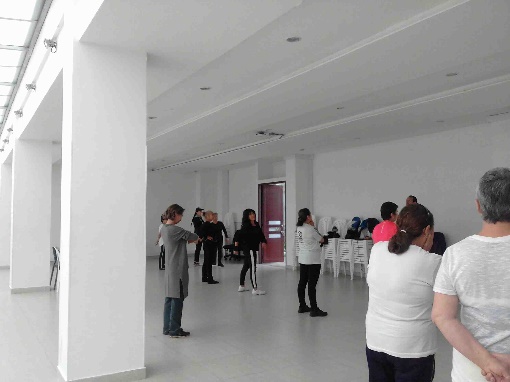 |
| Safety | "The park has very good lighting at night. It is also nice to come and share with the family, it has many lights, and they are all there. It's not dark, so it's very nice to be with family at night, and it's not dangerous at all." (My Body participant) | 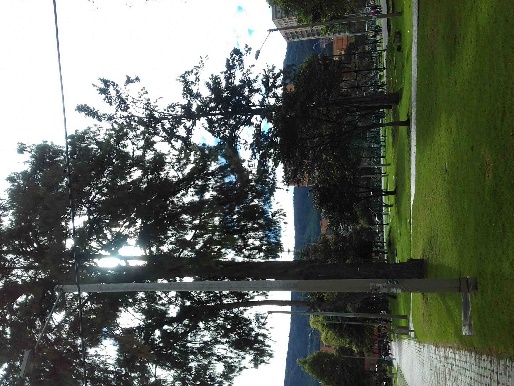 |
| Accessibility | "Well, I took this photo because it is one of the routes that make it easier for us to get to El Tunal, which is the place we chose, Paula, Ana and me, Maria, to be central. We studied the sectors and it is a central place so that we can all participate and continue with the physical activity, which has served us all a lot for our health." (My Body participant) | 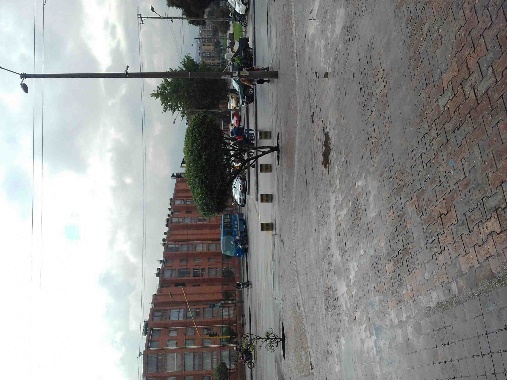 |
| Space logistics | "I really like this environment because it is big, but what I don't like is that there are always many vicious people around here. A lot of people who bring the animals to the park, and leave their feces watered. I would also like the park to be enclosed because it's safer that way." (My Body participant) | 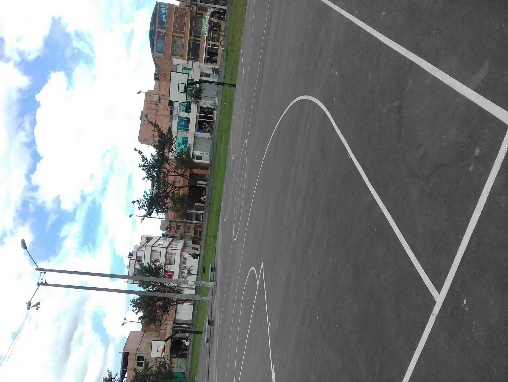 |
| Sanitation maintenance | "I like to exercise in this park because it's always very clean, and the community is very friendly." (My Body participant) | 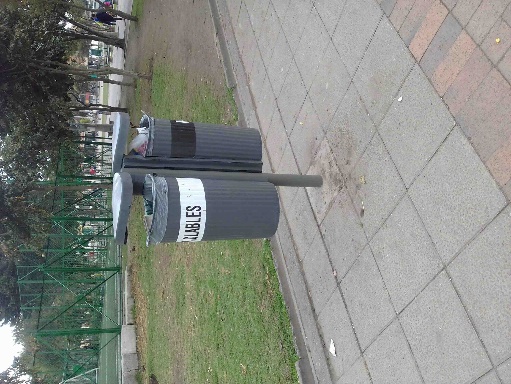 |
| Sense of community | "I highlight the importance that we can come here, with our pets, with our loved ones. Sometimes I come with my mommy, and we share a very nice moment. My mommy is already an old person, and so I share with her. First of all, as I repeat, it's very safe." (My Body participant) | 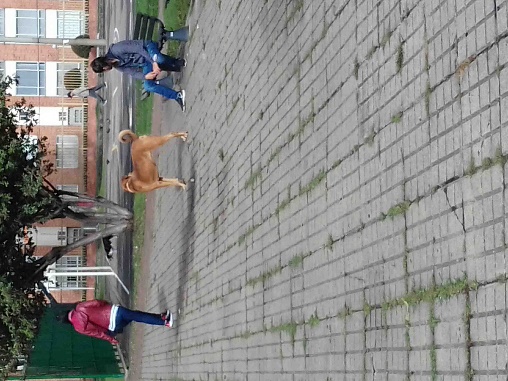 |
| Complementary services | "I take this picture because I think it's wonderful to have an accessible place where we can buy water to hydrate, suddenly get a snack, a fruit. Then I think it's very good." (My Body participant) | 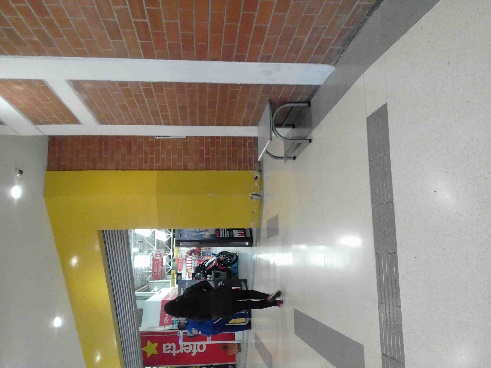 |
| Nature | "One of the facilitators I find is that there is a very harmonious part of the nature of this park, and it is very beautiful. Look at this point, on that side, the side of nature, it feels calm and harmonious." (My Body participant) | 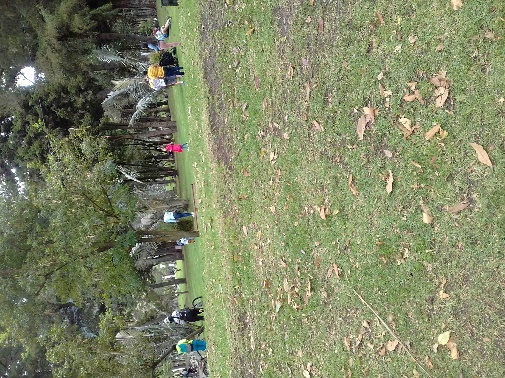 |
| Self-determination | "In this place at the moment I do not see barriers to continue doing physical activity, I am looking forward to continue and be able to do it here from 8 in the morning to 10."( My Body participant) | 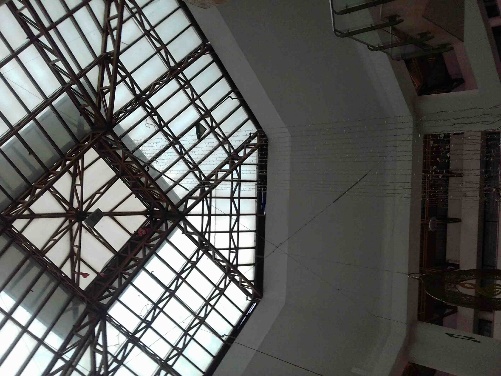 |
| Recreovía | "Well, I take this picture because one of the motivations, despite all the barriers, is the IDRD class, it's a rumba class that starts at 11:15, on Sundays and holidays. Well, I try to come on Sunday and this is the class I like the most, so that's the reason for the photo." (My Body participant) | 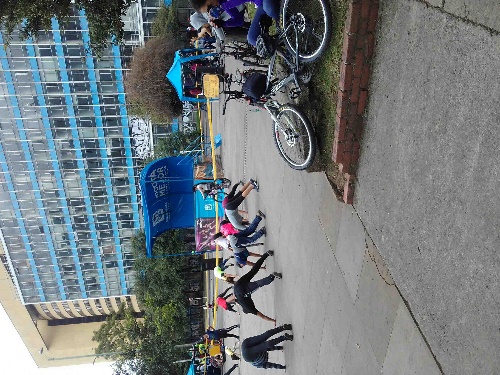 |
| Medical condition | "This is the space where the physical activity is carried out. I think this is excellent but maybe something that I see as a barrier is that the activities are really intense and they don’t really see our condition like they used to in the project we came from where they would take the necessary considerations regarding our state of health. Here everyone is treated the same and obviously they tell you to go at your own pace but we don’t have that priority, so to speak, where our diagnosis is taken into account or where we’re treated differently like they did in the project we came from.” (My Body participant) | 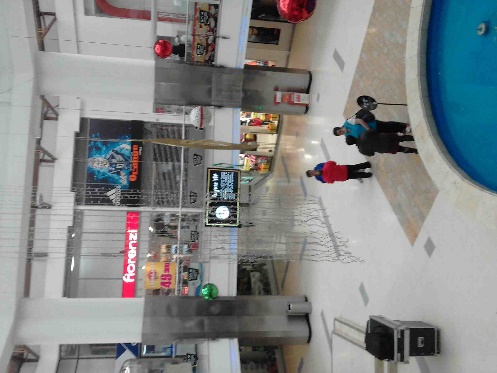 |
