# Supplementary material for: Innovative participatory evaluation methodologies to assess and sustain multilevel impacts of two community-based physical activity programs for women in Colombia
Source: BMC Public Health. 2022 Apr 15;22:771. doi: 10.1186/s12889-022-13180-2 (PMC9012256; doi:10.1186/s12889-022-13180-2)
Supplement: Supplementary file 2 — Additional file 2: Supplementary material 2. Ripple Effects Maps created by participants to report outcomes of the Recreovìa and My Body physical activity programs, respectively. [file 12889_2022_13180_MOESM2_ESM.pdf]

Supplementary material 2. Ripple effects maps created by participants of the Moving and My Body studies to report outcomes of the Recreovìa and My Body physical activity programs, respectively.

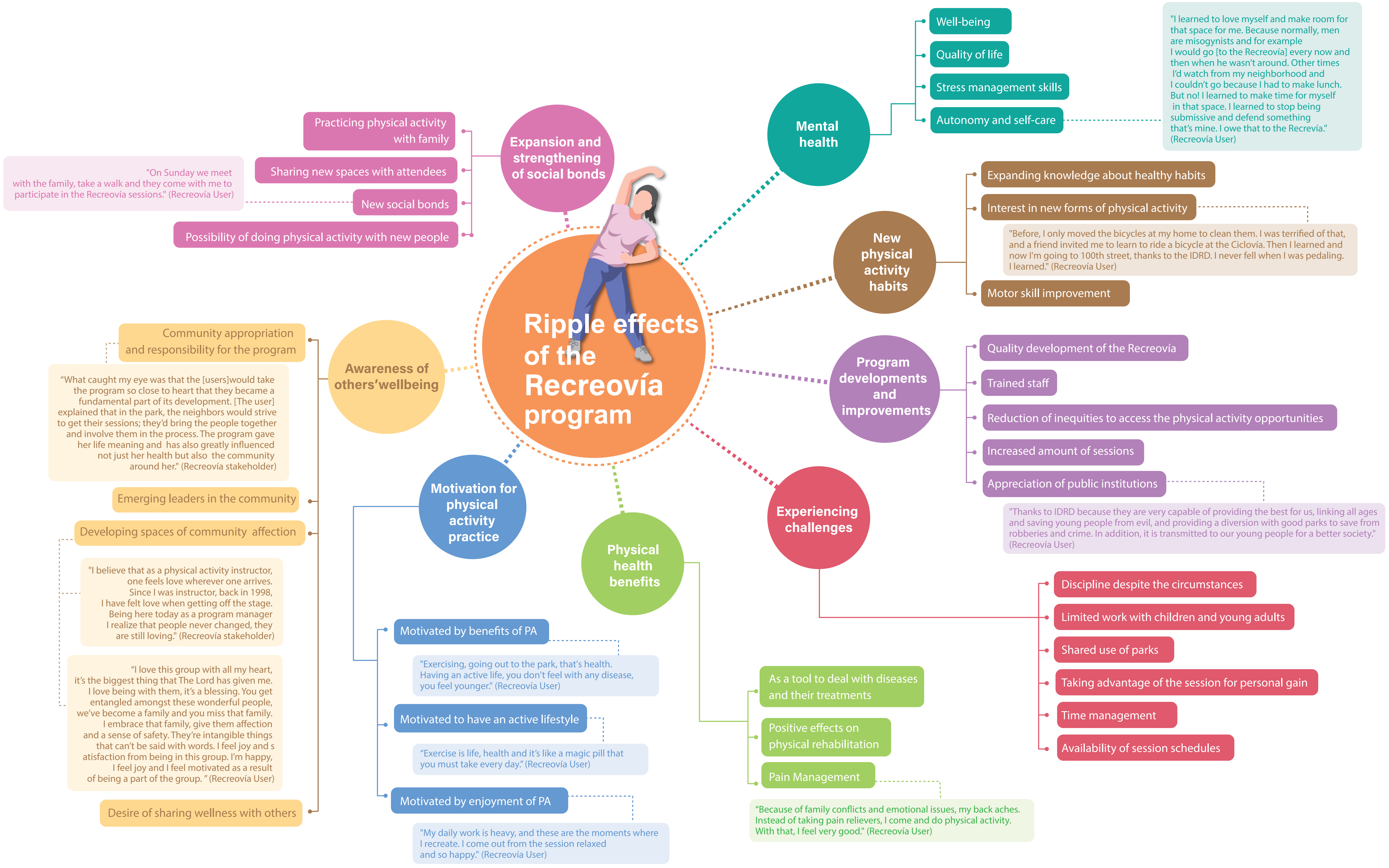

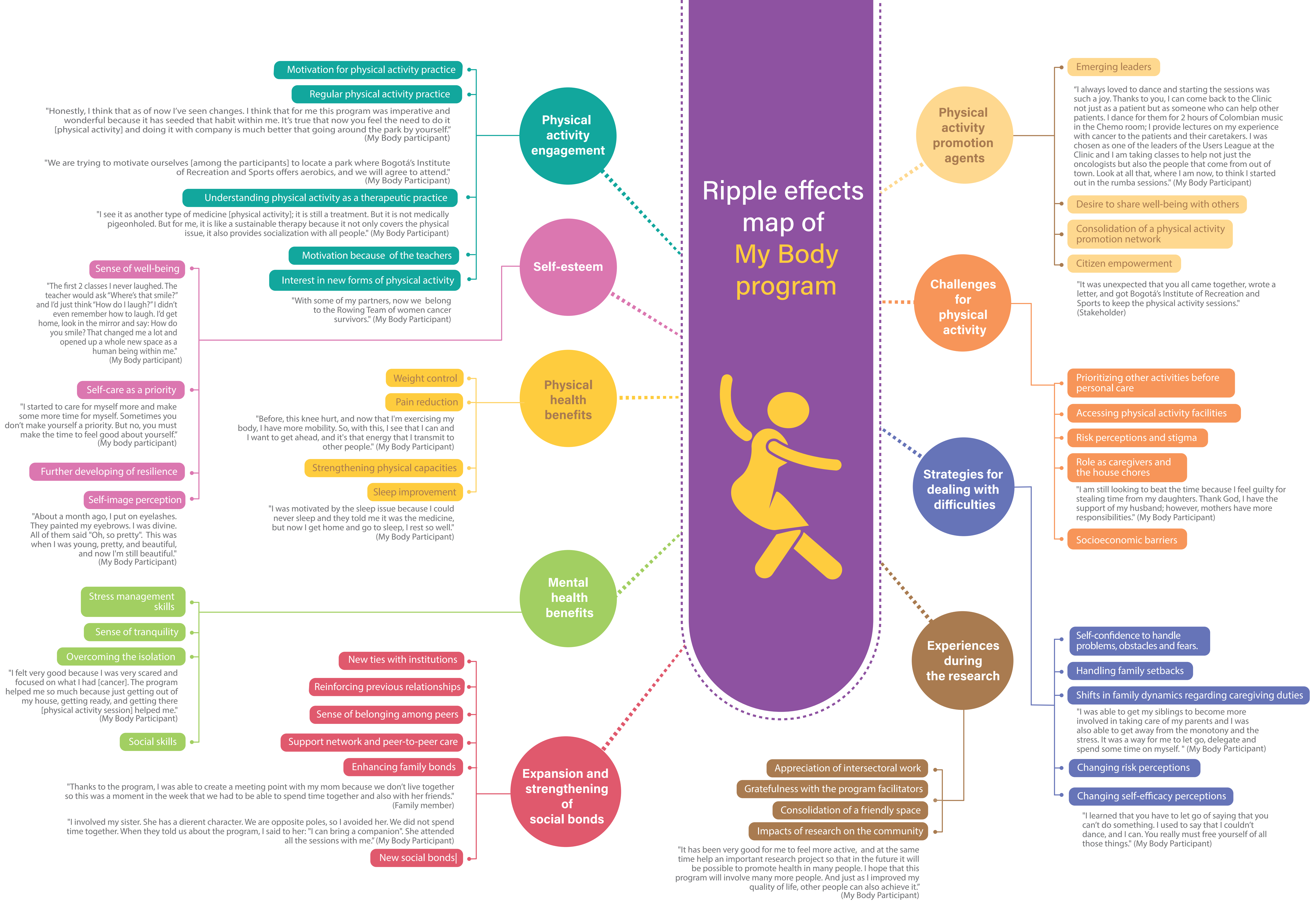

Ripple effects map of My Body program
Physical activity engagement
Motivation for physical activity practice
Regular physical activity practice
"Honestly, I think that as of now I've seen changes. I think that for me this program was imperative and wonderful because it has seeded that habit within me. It's true that now you feel the need to do it [physical activity] and doing it with company is much better that going around the park by yourself." (My Body participant)
"We are trying to motivate ourselves [among the participants] to locate a park where Bogotá's Institute of Recreation and Sports offers aerobics, and we will agree to attend." (My Body Participant)
Understanding physical activity as a therapeutic practice
"I see it as another type of medicine [physical activity]; it is still a treatment. But it is not medically pigeonholed. But for me, it is like a sustainable therapy because it not only covers the physical issue, it also provides socialization with all people." (My Body Participant)
Motivation because of the teachers
Interest in new forms of physical activity
"With some of my partners, now we belong to the Rowing Team of women cancer survivors." (My Body Participant)
Self-esteem
Sense of well-being
"The first 2 classes I never laughed. The teacher would ask "Where's that smile?" and I'd just think "How do I laugh?" I didn't even remember how to laugh. I'd get home, look in the mirror and say: How do you smile? That changed me a lot and opened up a whole new space as a human being within me." (My Body participant)
Self-care as a priority
"I started to care for myself more and make some more time for myself. Sometimes you don't make yourself a priority. But no, you must make the time to feel good about yourself." (My body participant)
Further developing of resilience
Self-image perception
"About a month ago, I put on eyelashes. They painted my eyebrows. I was divine. All of them said "Oh, so pretty". This was when I was young, pretty, and beautiful, and now I'm still beautiful." (My Body Participant)
Physical health benefits
Weight control
Pain reduction
"Before, this knee hurt, and now that I'm exercising my body, I have more mobility. So, with this, I see that I can and I want to get ahead, and it's that energy that I transmit to other people." (My Body Participant)
Strengthening physical capacities
Sleep improvement
"I was motivated by the sleep issue because I could never sleep and they told me it was the medicine, but now I get home and go to sleep, I rest so well." (My Body Participant)
Mental health benefits
Stress management skills
Sense of tranquility
Overcoming the isolation
"I felt very good because I was very scared and focused on what I had [cancer]. The program helped me so much because just getting out of my house, getting ready, and getting there [physical activity session] helped me." (My Body Participant)
Social skills
Expansion and strengthening of social bonds
New ties with institutions
Reinforcing previous relationships
Sense of belonging among peers
Support network and peer-to-peer care
Enhancing family bonds
"Thanks to the program, I was able to create a meeting point with my mom because we don't live together so this was a moment in the week that we had to be able to spend time together and also with her friends." (Family member)
"I involved my sister. She has a dierent character. We are opposite poles, so I avoided her. We did not spend time together. When they told us about the program, I said to her: "I can bring a companion". She attended all the sessions with me." (My Body Participant)
New social bonds|
Physical activity promotion agents
Emerging leaders
"I always loved to dance and starting the sessions was such a joy. Thanks to you, I can come back to the Clinic not just as a patient but as someone who can help other patients. I dance for them for 2 hours of Colombian music in the Chemo room; I provide lectures on my experience with cancer to the patients and their caretakers. I was chosen as one of the leaders of the Users League at the Clinic and I am taking classes to help not just the oncologists but also the people that come from out of town. Look at all that, where I am now, to think I started out in the rumba sessions." (My Body Participant)
Desire to share well-being with others
Consolidation of a physical activity promotion network
Citizen empowerment
"It was unexpected that you all came together, wrote a letter, and got Bogotá's Institute of Recreation and Sports to keep the physical activity sessions." (Stakeholder)
Challenges for physical activity
Prioritizing other activities before personal care
Accessing physical activity facilities
Risk perceptions and stigma
Role as caregivers and the house chores
"I am still looking to beat the time because I feel guilty for stealing time from my daughters. Thank God, I have the support of my husband; however, mothers have more responsibilities." (My Body Participant)
Socioeconomic barriers
Strategies for dealing with difficulties
Self-confidence to handle problems, obstacles and fears.
Handling family setbacks
Shifts in family dynamics regarding caregiving duties
"I was able to get my siblings to become more involved in taking care of my parents and I was also able to get away from the monotony and the stress. It was a way for me to let go, delegate and spend some time on myself. " (My Body Participant)
Changing risk perceptions
Changing self-efficacy perceptions
"I learned that you have to let go of saying that you can't do something. I used to say that I couldn't dance, and I can. You really must free yourself of all those things." (My Body Participant)
Experiences during the research
Appreciation of intersectoral work
Gratefulness with the program facilitators
Consolidation of a friendly space
Impacts of research on the community
"It has been very good for me to feel more active, and at the same time help an important research project so that in the future it will be possible to promote health in many people. I hope that this program will involve many more people. And just as I improved my quality of life, other people can also achieve it." (My Body Participant)
